# Transcriptome profiling reveals association of peripheral adipose tissue pathology with type-2 diabetes in Asian Indians

**DOI:** 10.1080/21623945.2019.1595269

**Published:** 2019-03-28

**Authors:** Aditya Saxena, Pradeep Tiwari, Nitin Wahi, Arpana Soni, Ram Chandra Bansiwal, Anshul Kumar, Balram Sharma, Poonam Punjabi, Nidhi Gupta, Babita Malik, Krishna Mohan Medicherla, Prashanth Suravajhala, Sandeep Kumar Mathur

**Affiliations:** a Department of Biotechnology, Institute of Applied Sciences and Humanities, GLA University, Mathura, India; b Department of Endocrinology, Sawai Man Singh Medical College and Hospital, Jaipur, India; c Department of Biotechnology and Bioinformatics, Birla Institute of Scientific Research (BISR), Jaipur, India; d Department of Chemistry, School of Basic Sciences, Manipal University, Jaipur, India; e Department of Biotechnology, The IIS University, Jaipur, India

**Keywords:** Type 2 diabetes, weighted gene co-expression network analysis, signalling pathway impact analysis, Asian Indians, peripheral adipose tissue, system biology

## Abstract

Type 2 diabetes (T2D) is a complex disease with an elusive link between its molecular aetiology and clinical presentation. Although, the role of visceral adipose tissue in insulin-resistance and T2D is known, limited information is available on the role of peripheral-subcutaneous adipose tissue especially in Asian Indians. In this microarray-based study of diabetic and normal glucose tolerant Asian Indians, we generated the transcriptome of their thigh adipose tissue and analyzed differentially expressed genes (DEGs) using weighted gene co-expression network analysis; further we identified perturbed pathways implicated by these DEGs in relevant co-expression modules. We also attempted to link these pathways with known aspects of T2D pathophysiology in terms of their association with some of their intermediate traits, namely; adipocyte size, HOMA-B, HOMA-R, Hb1Ac, insulin, glucose-level, TNF-α, IL-6, VLDLs, LDLs, HDLs, and NEFAs. It was observed that several modules of co-expressed genes show an association with diabetes and some of its intermediate phenotypic traits mentioned above. Therefore, these findings suggest a role of peripheral subcutaneous adipose tissue in the pathophsiology of T2D in Asian Indians. Additionally, our study indicated that the peripheral subcutaneous adipose tissue in diabetics shows pathologic changes characterized by adipocyte hypertrophy and up-regulation of inflammation-related pathways.

## Graphical Abstract

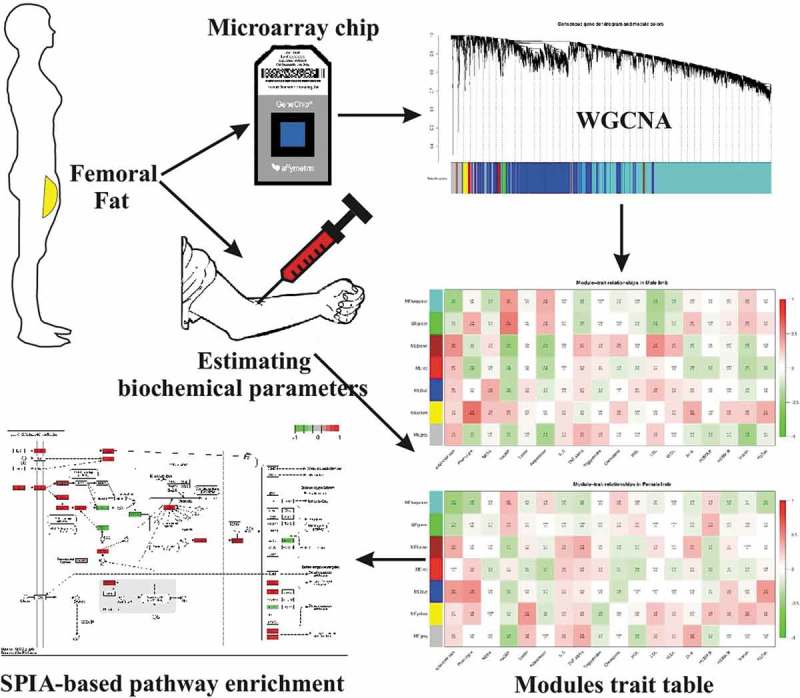

## Introduction

Type 2 diabetes mellitus (T2D), the most common subtype of diabetes is a disease characterized by hyperglycaemia and it arises from a varying combination of high resistance to action and relative deficiency of the hormone insulin [1]. The accumulation of excess fat, i.e. obesity has long been implicated in the development of insulin resistance with the subsequent clinical manifestation of type 2 diabetes and metabolic syndrome. However, Asian Indians have been found to be at a greater risk of developing these diseases even at lower body mass index [2]. This peculiar pathophysiological tendency has been attributed to their typical ‘thin fat’ phenotype—the accumulation of higher visceral fat (central obesity) due to relatively small peripheral fat compartment—also termed as ‘nutrient overflow hypothesis’ [3–5]. Moreover, they have also been reported to show low glucose disposal rate, high levels of plasma non-esterified fatty acids (NEFAs), hsCRP, leptin and lower levels of adiponectin than Caucasians even when corrected for excess body fat [6,7]. Therefore, these findings clearly point towards an additional qualitative defect in adipose tissue pathophgysiology where Asian Indians are genetically susceptible to the pathology of adipose tissue, the so-called *sick fat* or *adiposopathy* [8].

Earlier, we have reported the association of adiposopathy with T2D in visceral depot of Asian Indians by genome-scale transcriptome profiling [9]. However, the role of the peripheral fat in the T2D pathology remains dubious. Therefore, in the present study we have conducted a cross-sectional microarray-based transcriptome profiling of 30 normal glucose tolerants and, 30 T2D individuals from their thigh subcutaneous adipose tissue. Our hypothesis is that the inability of protective adipose tissue in places like the thigh to store excess calories leads to ectopic fat deposition in the liver and muscle [10] contributing to the initiation or progression of T2D. In this study, we have also measured adipocyte size and counted the numbers of infilterating macrophages (total, anti-inflammatory and pro-inflammatory) and lymphocytes in femoral fat as a likely marker of pathologic adipose tissue.

How the ‘sick fat’ or adiposopathy contributes to the pathophysiology of T2D and the link between molecular level changes and disease phenotypes remains elusive in T2D due to the complex interplay of various genetic, epigenetic and environmental factors. Previously, we addressed this issue by integrating microarray gene expression data of abdominal fat (both visceral, and subcutaneous) with other genome-scale data, viz. Genome Wide Association Studies, toxicogenomics, protein-protein interactions, gene-disease, etc., and successfully correlated certain aspects of the genome with T2D phenotypes [11]. Here, in this present study we have further attempted to establish this link by using a popular system biology method—weighted gene correlation network (*WGCNA*) approach [12] that has been used to identify modules of co-expressed genes; it also allows to relate these modules with external traits such as intermediate/biochemical traits. In this study, we have measured various biochemical parameters as intermediate traits of T2D viz. Homeostatic model assessment for β-cell secretion and insulin resistance (HOMA-B, and HOMA-R), glycated haemoglobin (Hb1Ac), blood insulin, glucose level, pro-inflammatory markers—TNF-α, IL-6, lipid profile markers—very low-density lipids (VLDLs), low-density lipids (LDLs), high-density lipids (HDLs) and non-esterified fatty acids (NEFAs) and shown their correlation with gene expression. Specifically, our analysis also indicated ‘adipocyte size’ as the most chronic cellular marker of adiposopathy, hence this parameter was also taken as intermediate phenotypic trait for correlation analysis with gene expression profiles. To sum up, the present study was designed (1) To ascertain pathology of peripheral adipose depot in T2D in terms of adipocyte size, infiltrating immune cells and transcription profile and (2) To identify co-expressed gene modules showing association with the disease itself and its intermediate phenotypic traits namely clinical diagnosis of diabetes, adipocyte size, homeostatic model assessment for β-cell secretion and insulin resistance (HOMA-B, and HOMA-R), glycated haemoglobin (Hb1Ac), blood insulin, glucose level, pro-inflammatory markers—TNF-α, IL-6, lipid profile markers—very low-density lipids (VLDLs), low-density lipids (LDLs), high-density lipids (HDLs), and non-esterified fatty acids (NEFAs).

## Material and methods

The study was conducted at Sawai Man Singh Medical College, Jaipur, India. A total 60 individuals with equal number of normal glucose tolerants (NGT) and T2D individuals (*n* = 30 in each group) with comparable sex ratio (M:F ratio 15:15 in each groups) undergoing femur bone surgery (neck and shaft of femur) for traumatic fracture were included in the study. The case:control ratio was 1:1 that was deemed as appropriate as per the ethical and clinical considerations in research on human subjects. The sex ratio was also 1:1 to avoid any confounding effect of gender. Our sample size (i.e. 60) was also in agreement with the recommendation of authors of WGCNA programme (https://horvath.genetics.ucla.edu/html/CoexpressionNetwork/Rpackages/WGCNA/faq.html). The study was approved by the ethical committee of SMS Medical College, Jaipur, India, and funded by the Indian Council of Medical Research (ICMR), New Delhi. Written informed consent before participation of subjects was obtained. All methods were performed in accordance with the relevant guidelines and regulations.

The inclusion and exclusion criteria for control and case subjects with variables have been defined appropriately (Table 1). Several factors like age, sex, menopausal status, obesity, trauma, drugs and diseases like inflammation and malignancy could potentially affect adipose tissue transcriptome and could lead to a bias in the design of the study. Following steps were taken to overcome these biases: (1) Both NGT and T2D groups were matched for age and sex. (2) The gluteofemoral adipose tissue is known to protect from the development of insulin resistance and metabolic syndrome in reproductive age women but it is probably no more protective in post-menopausal women. Hence, the potential bias due to menopausal status was avoided by adopting age cut off of >50 years, where most of NGT and T2DM were post menopausal. (3) Direct trauma can induce adipose tissue inflammation and alter adipose tissue transcriptome. In the present study, the site for biopsy was the site of surgical incision, which was in most cases different from the site of trauma. Moreover, the indications for the surgery in both the cases and control groups were similar. Therefore, the possible bias of the direct trauma or surgery indication on the eventual result of the study was nullified. (4) Obesity is known to be associated with pathologic changes in adipose tissue transcriptome; therefore, we included non-obese individuals in this study and the cut-off BMI opted was less than 30 kg/m^2^.Although in the Asian population BMI < 27.5 kg/m^2^ has been proposed as the cut-off for obesity but this cut-off is yet to be validated in the western Indian population being studied here. Thus, for the purpose of this study we chose 30 kg/m^2^ as the cut-off for obesity. However, still the mean BMI for NGT and T2D groups was less than 27.5 kg/m^2^; therefore, negating the potential bias that may arise due to the contentious obesity associated adipose tissue pathology in this gene expression study. (5) As mentioned in the exclusion criteria, infection, malignancy and drugs affecting adipose tissue were ruled out before inclusion in this study.

**Table 1.** Inclusion and exclusion criteria for the study subjects.

Condition	Inclusion criteria	Exclusion criteria
Diabetic subjects	Non-obese (BMI < 30) type-2 diabetics diagnosed as per American Diabetes Association (ADA, 2012) criteria undergoing femur bone surgery.	Presence of infection, malignancy and drugs affecting body fat/insulin resistance or adipokine expression like glitazones, metformin and glucocorticoids.
Non-diabetic	Age and sex-matched non-obese (BMI < 30) normal glucose tolerance subjects undergoing femur bone surgery.	Presence of infection, malignancy and drugs affecting body fat/insulin resistance or adipocytokines expression like glitazones, metformin and glucocorticoids. History of diabetes in first degree relatives.

### Assessment of biochemical parameters

Various biochemical parameters like serum glucose, lipid profile, triglycerides, LDL, HDL and VLDL were measured on Kopran AU/400 fully automated analyzer. Serum insulin was measured using a chemiluminescent immunometric assay (Immulite 2000 machine) (senstivity—0.5 mU/l, CV%—7.5). HbA1c was measured by turbidimetry method using kits supplied by BioSystems. Insulin resistance was calculated by HOMA-IR and HOMA-β was used to measure β-cell function. NEFAs were measured by the biochemical method (Randox Laboratories Ltd UK kits) (senstivity—0.072 mmol/l, CV%—4.74). High sensitivity C reactive protein (hsCRP, Diagnostics Biocheme Canada Inc, Canada) (senstivity—10 ng/ml, CV%—7.8), Leptin (Lab systems Diagnostic Oy, Finland) (senstivity—<62.5 pg/ml, CV%—<12), adiponectin (Lab systems Diagnostic Oy, Finland) (senstivity—0.185 ng/ml, CV%—<10), Il-6 (Lab systems Diagnostic Oy, Finland), TNF-α (Lab systems Diagnostic Oy, Finland) was estimated by ELISA method. Body fat content of patients undergoing femur surgery was estimated by dual-energy X-ray absorptiometry (DXA) using Hologic Explorer model (S/N91395 make) (see supplementary File S1 for measured biochemical parameters).

### Microarray data collection

Transcription profiling for 60 samples, viz. 30 controls and 30 diabetic subjects was performed to study the expression level of genes from the thigh subcutaneous adipose tissues (GEO Accession # GSE78721). Total RNA from each thigh biopsy sample was isolated using QiagenRNeasy Mini Kit (Cat No. 74104). The quantification of samples was done using a Microfluidic-based capillary electrophoresis system (Bio-Rad Experion). The transcriptome Analysis of the sample was done using Affymetrix GeneChip PrimeView Human Gene Expression Array. Total RNA was made to undergo reverse transcription to synthesize the first-strand cDNA. The cDNA, thus formed was then converted to double-stranded cDNA template during second-strand cDNA synthesis. Biotinylated ribonucleotide was then incorporated by *in vitro* transcription reaction and was then purified by bead-based purification method. The purified biotin-labelled-cRNA was then fragmented using a fragmentation buffer and then the sample was hybridized onto GeneChip 3ʹ expression array.

### Quantitative PCR (qPCR)

A total of 2 µg of RNA was isolated from the thigh adipose tissue using QiagenRNeasy Mini Kit (Cat No. 74104) and quantified using the microfluidic-based capillary electrophoresis system (Bio-Rad Experion). cDNA synthesis was done using the QuantiNova Reverse Transcription Kit (Cat No. 205411), and quantitative PCR was done using the QuantiNova Probe PCR Kit (Cat. No 208252). The oligonucleotide primer sequences used in the qPCR analysis were as listed in Table 2. qPCR expression values for all the genes were found to be consistent with the direction of gene expression between normal and type 2 diabetic samples in the microarray experiment (see supplementary File S2).

**Table 2.** PCR primer-pairs used in qPCR.

S. No.	Gene	Primer pair
1	*ISG15*	Forward, 5ʹ- GAGGCAGCGACTCATCTTT-3ʹ and Reverse, 5ʹ-AGCATCTTCACCGTCAGGTC-3’
2	*VWA1*	Forward, 5ʹ-TCTCGCTAGGATGCTTTACTCC-3ʹ and Reverse, 5ʹ-CGTTCACAAGCTGTCCTGTC-3’
3	SOS1	Forward, 5ʹ-CAAGTTCCCCCTAATTTGACAT-3ʹ and Reverse, 5ʹ-CAACCTCCTCCCCCATAATAA-3’
4	*SLC33A1*	Forward, 5ʹ-CCTTGAATCTGCCGACTTTT-3ʹ and Reverse, 5ʹ-TGTTATTAAAAATACAGTTCCCCAGA-3’
5	*MUSTN1*	Forward, 5ʹ-GGCTCCACTCAGATCTTTTCC-3ʹ and Reverse, 5ʹ-ATAGGGGCTTCCTGAGCAC-3’
6	*ZNF638*	Forward, 5ʹ-CCAAGGTGATCTCACACTCCT-3ʹ and Reverse, 5ʹ-AAACCATGTTCTTTGTTTGTTTAAGA-3’
7	*CTIF*	Forward, 5ʹ-GCGAAGGCTAAAGGAAAAGG-3ʹ and Reverse, 5ʹ-TCAGGATCTCGATCAGCTTG-3’
8	*ACTB* (housekeeping gene)	Forward, 5ʹ-CCAACCGCGAGAAGATGA-3ʹ and Reverse, 5ʹ-CCAGAGGCGTACAGGGATAG-3’
9	*G6PD* (housekeeping gene)	Forward, 5ʹ-AACAGAGTGAGCCCTTCTTCA-3ʹ and Reverse, 5ʹ-GGAGGCTGCATCATCGTACT-3ʹ.

### Bioinformatics analysis

Low-level analysis of microarray datasets was carried out using Bioconductor packages: *gcrma, genefilter* and *org.Hs.eg.db* separately for both genders. As the annotation package for the prime view was not available, it was created using *human.db0*, and *AnnotationForge* package using prime-view annotation file and gender-wise differentially expressed (DE) genes between NGT and T2D were estimated using *limma* method. Gender-wise DE genes were then imported to ClueGO app [13] for network-based visualization of enriched pathways (*P < 0.05*) (see supplementary File S3). For *WGCNA* analysis, first a multi-set expression data object was created from normalized expression values of male and female datasets and checked for an excessive number of missing samples. The samples in the multi-set expression data were matched with the corresponding physiological traits. A one-step consensus network construction and module detection was then used by selecting soft thresholding power β = 15. The value of β ensures that co-expression network thus formed will follow the scale-free topology and hence can be considered as biologically more plausible. In this way, we obtained seven consensus modules (see Supplementary File S4). To relate these consensus modules with the physiological traits, *WGCNA* provides the function to estimate module eigengenes and then relate the clinical data with the modules using correlation. To summarize the two sets into one measure, we took the correlation that has the lower absolute value in the two sets if the two correlations have the same sign, and zero relationship if the two correlations have opposite signs. Furthermore, we evaluated the absolute correlation of each gene for all the intermediate traits—termed as Gene Significance (GS) both in term of correlation and *P* value.

Consensus modules showing statistically significant correlation with measured physiological traits were further analyzed for enrichment of cellular pathways using a sophisticated method, signalling pathway impact analysis (*SPIA*) [14]. This method combines two types of evidences: (i) The overrepresentation of DE genes in a given pathway (*P^NDE^*) and (ii) The abnormal perturbation of that pathway as measured by propagating measured expression changes across the pathway topology (*P^PERT^*). All the 55 signalling pathways from KEGG were manually downloaded in KGML format and were used as input in SPIA programme along with genes and their log fold changes in the *turquoise, blue, yellow* and *brown* module.

## Results

### Anthropometric and biochemical characteristics

The values of Hb1Ac, HOMA-R, and insulin were found to be statistically extremely significant between NGT and T2D groups (in both male and female). Diabetic females had significantly higher leptin levels than the corresponding controls. There was no significant difference in the levels of other adipo-cytokines between diabetics and controls of both genders. Female diabetics had a higher total body and regional fat content than controls. Male diabetics had higher total body lean mass than controls. Table 3 presents gender-wise measured anthropometric, and biochemical parameters.

**Table 3.** Gender-wise measured anthropometric, and biochemical parameters in both NGT and T2D groups (expressed as means ± SD).

Gender	Female			Male		
Group	NGT	T2D	t test	NGT	T2D	t test
Age	65.80 ± 13.64	64.12 ± 8.15	0.65	61.95 ± 9.07	58.87 ± 9.17	0.33
Weight (kg)	52.10 ± 10.17	63.12 ± 16.06	0.02	55.80 ± 6.92	63.07 ± 11.02	0.04
BMI	21.49 ± 4.02	26.31 ± 6.96	0.02	20.28 ± 2.80	22.73 ± 3.25	0.03
W: H	0.97 ± 0.07	0.99 ± 0.10	0.50	1.03 ± 0.21	1.03 ± 0.07	0.90
Fasting glucose (mg/dl)	90.42 ± 12.48	183.32 ± 72.45	0.00007	88.96 ± 9.69	204.36 ± 102.13	0.0006
Triglyceride	163.70 ± 79.26	154.65 ± 53.17	0.68	154.32 ± 44.02	144.57 ± 55.02	0.59
Total cholesterol	195.60 ± 42.19	182.04 ± 35.58	0.30	180.65 ± 37.41	192.63 ± 66.90	0.56
HDL	40.90 ± 8.83	41.61 ± 4.00	0.75	40.47 ± 4.43	40.21 ± 7.62	0.91
LDL	87.39 ± 20.63	93.05 ± 21.91	0.43	94.28 ± 19.16	95.69 ± 33.11	0.89
VLDL	31.71 ± 8.44	51.09 ± 40.88	0.07	31.22 ± 6.79	33.92 ± 10.65	0.44
Serum creatinine	1.04 ± 0.32	1.04 ± 0.38	1.00	0.99 ± 0.17	1.08 ± 0.20	0.22
HOMA-B	192.89 ± 252.9	115.68 ± 125.97	0.24	132.87 ± 87.65	126.59 ± 126.75	0.87
HOMA-R	2.45 ± 2.13	12.30 ± 11.38	0.002 *P* val. = 0.002667	1.83 ± 1.47	11.97 ± 7.16	0.00007 *P val*. = 7.528e-05
Insulin	11.16 ± 9.89	26.22 ± 19.00	0.01 *P* val. = 0.007192	8.25 ± 5.93	24.44 ± 10.77	0.00003 *P val.* = 3.627e-05
Hb1Ac (%)	5.45 ± 0.62	8.17 ± 1.38	0.0000002 *P* val. = 1.912e-07	5.32 ± 0.55	9.22 ± 2.75	0.00007 *P val*. = 7.662e-05
NEFA (mmol/L)	0.62 ± 0.30	0.63 ± 0.48	0.92	0.51 ± 0.26	0.66 ± 0.43	0.26
HsCRP (ng/ml)	7950.78 ± 5701.0	8769.12 ± 4779.9	0.64	7279.16 ± 5140.79	10202.27 ± 4134.99	0.07
Leptin (pg/ml)	8150.77 ± 7945.67	19144.99 ± 27723.65	0.13	7810.80 ± 5691.17	8094.73 ± 6584.18	0.89
Adiponectin (ng/ml)	210.71 ± 116.19	236.74 ± 239.48	0.69	183.76 ± 117.78	208.99 ± 86.46	0.47
IL-6	33.33 ± 53.05	30.53 ± 33.71	0.85	22.02 ± 29.45	30.34 ± 46.60	0.55
TNF-α	25.42 ± 17.51	28.84 ± 35.04	0.72	32.70 ± 29.63	28.75 ± 35.79	0.73

### Adipocyte cell size and infiltrating immune cells

The diabetic females had significantly higher adipocyte size, and it was almost double than the controls. (Table 4). There was no statistically significant difference between diabetics and NGT controls of both the genders in the number of infiltrating inflammatory cells and cell (Table 5). These results indicate that adipocyte hypertrophy is a major cellular pathologic feature of femoral subcutaneous adipose tissue in diabetics and there was no consistent pattern of immune cell infiltration.

**Table 4.** Gender-wise comparison of adipocyte cell size in both NGT and T2D groups.

Male					Female				
Diabetics		Non-diabetic			Diabetics		Non-diabetic		
Mean	SD	Mean	SD	T test	Mean	SD	Mean	SD	T test
75873.3	47100.8	72550.4	35,412	0.82642	134491	67161.7	76443.2	48055.2	0.00593

**Table 5.** Gender-wise measured cell infiltration parameters in both NGT and T2D groups.

Cell infiltration data										
Antibody marker	Male					Female				
	Diabetics		Non-diabetic		T test	Diabetics		Non-diabetic		T test
	Mean	SD	Mean	SD		Mean	SD	Mean	SD	
CD 44	4.78	5.76	1.4	2.67	0.19	0.39	0.69	2.5	4.47	0.14
CD 163	8.77	7.28	3.31	2.37	0.1	4.25	5.44	8.26	10.92	0.33
CD 3	2.54	2.7	0.61	0.66	0.11	0.8	0.76	1.36	1.69	0.36
HAM 56	0.68	0.79	1.56	2.38	0.3	0.53	0.37	1.28	2.44	0.32

## Gene expression analysis

A total of 971 DE genes were found between NGT and T2D; however, gender specific DE analysis between these groups entails that there exists a marked influence of gender in the T2D pathophysiology (*DE_F_: 2949; DE_M_: 335*).

Gender-wise ClueGO pathway enrichment (*P < 0.05*) for DE genes revealed various immune system-related term-leading pathways: *Class I Mediated antigen processing and presentation, neutrophil degranulation, CLEC7A (Dectine-1) signalling, Interleukin 10 signalling, IL1 and megakaryocytes in obesity, Staphylococcus aureus infection* and *amoebiasis*. These results conspicuously indicate that thigh adipose compartment is indeed inflamed in diabetics much like its abdominal counterpart. Furthermore, female adipose tissue has also showed enrichment of various RNA, and protein metabolism as well as vesicle-mediated transport-related pathways such as *translation, protein processing in endoplasmic reticulum, RNA transport, trans-Golgi network vesicle budding* and *membrane trafficking*, leading to the conclusion that female compartment is physiologically more active than their male counterpart.

**Figure 1.**
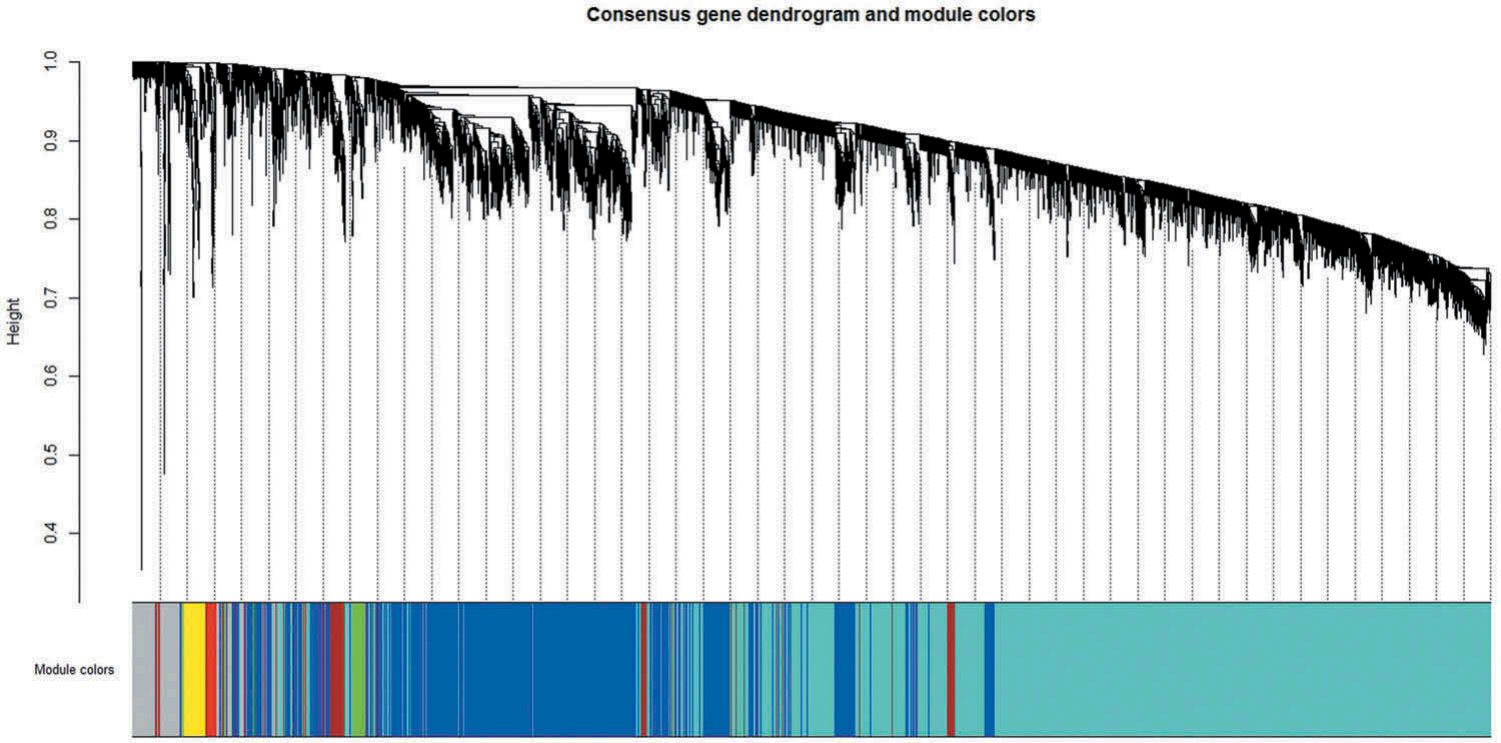
Consensus gene dendrogram with seven modules constructed using expression profiles of 7186 genes from male and female microarray datasets in the network.

**Figure 2.**
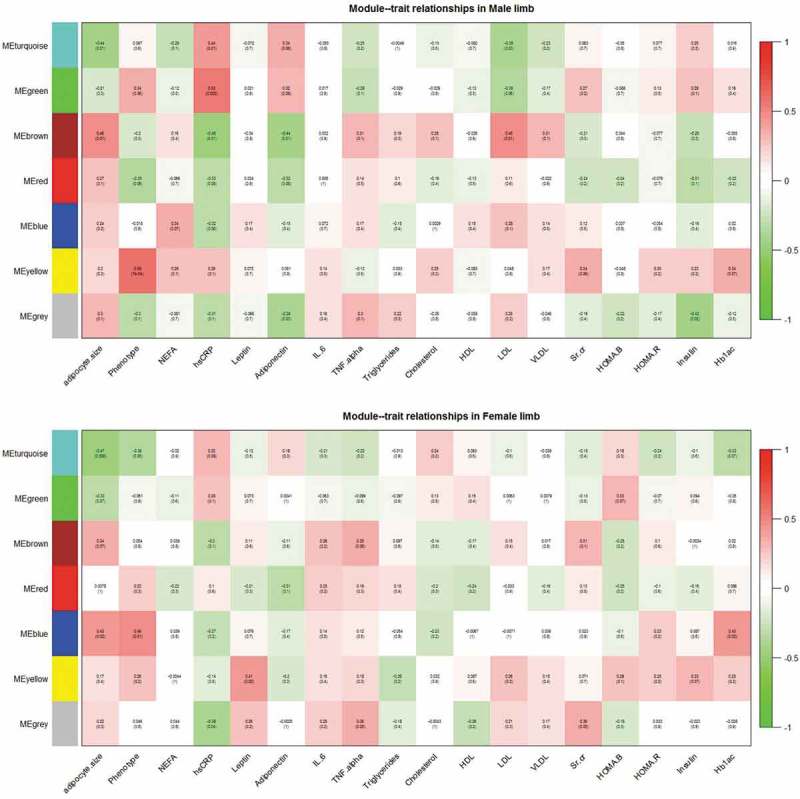
Module-trait relationships in the male and female limb datasets.

Subsequently, *WGCNA* was carried out separately on both the datasets and seven consensus modules of co-expressed genes were obtained: turquoise, green, brown, red, blue, yellow and grey (Figure 1). Gene modules showing statistically significant correlation (*P < 0.05)* with gender-wise intermediate traits were identified (Figure 2) and their percentage DE gene enrichment was calculated. (Table 6) On the basis of numbers of correlated traits and genes, three modules were selected for SPIA-based functional analysis against human subset of KEGG signalling pathways.

**Table 6.** Consensus modules showing correlation (*P < 0.05*) with intermediate traits.

			Male		Female	
S. No.	Name of consensus module	Total nodes (genes)	% of genes mapped as differentially regulated	Modules showing correlation (*P < 0.05*) with intermediate traits	% of genes mapped as differentially regulated	Modules showing correlation (*P < 0.05*) with intermediate traits
1	Turquoise	4058	20.3	Adipocyte size, hsCRP, LDL	48.8	Adipocyte size, phenotype
2	Green	77	7.2	hsCRP	0.1	–
3	Brown	329	8.1	Adipocyte size, hsCRP, Adiponectin, LDL	0.9	–
4	Red	53	7.5	–	0.2	–
5	Blue	2212	11.0	–	46.9	Adipocyte size, phenotype, Hb1Ac
6	Yellow	120	31.0	Disease status (NGT vs T2D)	0.5	Leptin
7	Grey	337	14.9	Adiponectin, insulin	2.5	hsCRP, TNF-α, Sr.Cr.

Table 7 shows the top 20 affected pathways in *turquoise* module that comprise of maximum number of DE genes in both the datasets.

**Table 7.** Top 20 perturbed KEGG signalling pathways implicated by genes in turquoise module.

S. No.	Pathway	pERT	pG	Status
1	HIF-1 signalling pathway	5.00E-06	6.60E-05	Activated
2	Toll-like receptor signalling pathway	0.001	0.007908	Activated
3	cGMP-PKG signalling pathway	0.009	0.051395	Activated
4	NF-kappa B signalling pathway	0.009	0.051395	Activated
5	p53 signalling pathway	0.009	0.051395	Inhibited
6	AMPK signalling pathway	0.015	0.077996	Activated
7	Relaxin signalling pathway	0.027	0.124522	Activated
8	Estrogen signalling pathway	0.037	0.158983	Activated
9	NOD-like receptor signalling pathway	0.038	0.162266	Activated
10	Hippo signalling pathway	0.043	0.178302	Inhibited
11	AGE-RAGE signalling pathway in diabetic complications	0.043	0.178302	Activated
12	Apelin signalling pathway	0.058	0.223144	Activated
13	GnRH signalling pathway	0.075	0.26927	Activated
14	IL-17 signalling pathway	0.077	0.274424	Activated
15	Sphingolipid signalling pathway	0.082	0.287085	Activated
16	TGF-beta signalling pathway	0.087	0.299441	Inhibited
17	Phospholipase D signalling pathway	0.088	0.301877	Activated
18	mTOR signalling pathway	0.091	0.309118	Activated
19	Insulin signalling pathway	0.091	0.309118	Activated
20	Adipocytokine signalling pathway	0.565	0.887575	Activated

The literature search revealed associations of many of these pathways with known aspects of adipose tissue-related diseases. The role of *the HIF-1 signalling pathway* in adipose physiology has been documented and it is mediated by a transcription factor, hypoxia-inducible factor (HIF), whose high expression has been found in obesity-induced hypoxic conditions and may be the causative factor for adipose tissue inflammation [15]. Pharmacological intervention of this pathway may therefore, have the potential to ameliorate adipose functions.

Furthermore, over-expression of the receptor for advanced glycation end products (RAGE) has also been reported to cause adipocyte hypertrophy and suppression of insulin-mediated glucose uptake in cultured 3T3-L1 cells, thus linking the *AGE-RAGE signalling pathway in diabetes complications* with adipogenesis [16].

It is interesting to note that HIF-1 in fact, triggers the process of angiogenesis by promoting transcription of vascular endothelial cell growth factor (VEGF), stromal derived factor-1 (SDF-1) and platelet derived growth factor (PDGF) to alleviate tissue hypoxia. However, pronounced activation of AGEs and AGE receptors in diabetes contributes to impaired angiogenic potential [17]. Another pathway *Toll-like receptor signalling pathway* has been reported to link innate immunity with metabolism. Adipocytes do have a functional TLR4 system, whose activation by lipopolysaccharides and fatty acids leads to enhanced lipolysis and has been observed in metabolic alterations such as insulin resistance and T2D [18]. In addition, *cyclic guanosine monophosphate (cGMP)* signalling is known to enhance adipocyte differentiation and browning of white fat and one study has confirmed its deregulation in obesity-induced inflammation of visceral fat [19]. The activation of *p53* signalling pathway has been observed in mice fed with a high-fat diet and it has been argued that heightened lipolysis causes oxidative stress in adipocyte that leads to DNA damage and thus, could be the causative factor of adipose tissue inflammation and dysfunction [20].

Most of the metabolic effects of insulin signalling are mediated by *the PI3K-Akt pathway* that eventually leads to activation of IKK-NF/κB kinases and these kinases in turn, activate inflammatory *NF-kappa B signalling pathway* that in normal physiological conditions attenuates ‘Insulin signalling’ and thus establishes a negative feedback loop. However, in conditions of adiposopathy, heightened activation of this pathway results in insulin resistance with subsequent diabetes complications [21].

Activation of *AMPK signalling pathway* acts as a sensor of cellular energy status, and inhibition of pre-adipocyte differentiation and adipogenesis through increased phosphorylation of its substrate, acetyl-CoA carboxylase (ACC) [22]. Interestingly, a widely prescribed drug for type 2 diabetes—metformin inhibits hepatic gluconeogenic programme by activation of AMPK pathway [23] long-term usage of which may, therefore, have negative consequences on normal adipose physiology.

*Adipocytokine signalling pathway* is also relevant to adipogenesis as this pathway depicts the role of three adipocytokines—leptin, adiponectin and TNF-α in the process of insulin signalling. Decreased adiposity in peripheral fat is associated with low leptin and TNF-α levels but high adiponectin levels. Our consensus-*WGCNA* also reported *turquoise module* to be negatively correlated with leptin and TNF-α level but positively correlated with adiponectin level.

The other three significant modules, in term of percentage of DE genes and the number of correlated traits—*Blue, yellow* and *brown* were also evaluated for top 10 enriched signalling pathways are shown in Table 8.

**Table 8.** Top 10 perturbed KEGG pathways implicated by genes in blue, yellow and brown modules.

S. No.	Pathway	pERT	pG	Status
**Blue module**				
1	Ras signalling pathway	0.004	0.026086	Inhibited
2	Hedgehog signalling pathway	0.011	0.060608	Inhibited
3	PI3K-Akt signalling pathway	0.016	0.082163	Inhibited
4	IL-17 signalling pathway	0.022	0.105968	Inhibited
5	Chemokine signalling pathway	0.048	0.193755	Inhibited
6	Relaxin signalling pathway	0.088	0.301877	Inhibited
7	Neurotrophin signalling pathway	0.095	0.318618	Activated
8	Thyroid hormone signalling pathway	0.11	0.3528	Inhibited
9	Adrenergic signalling in cardiomyocytes	0.114	0.361557	Activated
10	Apelin signalling pathway	0.116	0.365883	Inhibited
**Yellow module**				
1	Wnt signalling pathway	0.117	0.368033	Inhibited
2	Hippo signalling pathway	0.576	0.893749	Inhibited
3	Rap1 signalling pathway	0.61	0.911521	Inhibited
4	mTOR signalling pathway	0.667	0.937112	Inhibited
5	MAPK signalling pathway	0.823	0.98332	Activated
6	Ras signalling pathway	0.847	0.987648	Activated
7	AGE-RAGE signalling pathway in diabetic complications	0.965	0.99938	Activated
8	TGF-beta signalling pathway	0.967	0.999449	Activated
9	PI3K-Akt signalling pathway	0.979	0.999778	Activated
10	FoxO signalling pathway	0.984	0.999871	Activated
**Brown module**				
1	IL-17 signalling pathway	0.019	0.094303	Inhibited
2	Toll-like receptor signalling pathway	0.026	0.120891	Inhibited
3	PI3K-Akt signalling pathway	0.04	0.168755	Inhibited
4	Prolactin signalling pathway	0.159	0.451377	Activated
5	Wnt signalling pathway	0.167	0.46589	Activated
6	Calcium signalling pathway	0.17	0.471233	Activated
7	Adipocytokine signalling pathway	0.174	0.478274	Activated
8	T cell receptor signalling pathway	0.283	0.640233	Inhibited
9	NOD-like receptor signalling pathway	0.311	0.674236	Activated
10	Epithelial cell signalling in Helicobacter pylori infection	0.32	0.684619	Inhibited

These modules enriched pathways related to immune system/inflammation (*IL-17 signalling pathway, Chemokine signalling pathway, Toll-like receptor signalling pathway, T cell receptor signalling pathway, NOD-like receptor signalling pathway* and *epithelial cell signalling in Helicobacter pylori infection*), cell division and differentiation-related pathways (*Ras signalling pathway, Hedgehog signalling pathway, Wnt signalling pathway and Hippo signalling pathway*) and various metabolic pathways that were well-documented downstream of insulin signalling (*mTOR signalling pathway, MAPK signalling pathway, Ras signalling pathway, AGE-RAGE signalling pathway in diabetic complications, TGF-beta signalling pathway, PI3K-Akt signalling pathway, Adipocytokine signalling pathway* and *FoxO signalling pathway*). We speculate that these modules contain genes that are related to physiological roles of insulin signalling however, working into a milieu of adiposopathy. *Hedgehog signalling pathway* has been reported to induce anti-adipogenic transcription factors upstream of PPARγ such as Gata2 in 3T3-L1 adipocytes and thus inhibit adipogenesis [24]. Similarly, *Wntsignalling pathway* has been ascribed to repress adipogenesis by blocking induction of PPARγ and CEBPA [25]. The role of *Hippo signalling pathway* in adipose biology has also been deciphered, and it was reported that Lats2, one of the core kinases of the Hippo pathway, inhibits proliferation of 3T3-L1 cells but promotes their differentiation through PPARγ-mediated transcription programme [26]. We opined that pharmacological inhibition of these pathways might provide valuable opportunities for diabetic management. *MAPK Signalling Pathway* and *PI3K-Akt signalling pathway* have been well studied downstream of insulin receptor and therefore have relevance in T2D pathology. While, the former mediates most of the anabolic effects of insulin signalling such as cell proliferation, differentiation, and gene expression, it is *PI3K-Akt signalling pathway* that has been ascribed to various metabolic effects of insulin signalling.

Besides modules of co-expressed genes and their correlations with intermediate traits, *WGCNA* also allows the estimation of gene significance (GS) for each gene in terms of all the measured external traits and hence offers a method of gene selection to be used as diagnostic/disease marker. We have estimated GS of all the 7,186 genes used in *WGCNA* for every intermediate trait (Supplementary File S3) and identified the top 20 genes (*HOXB3, RSPO3, HOXA5, GREM1, ORMDL1, C7, TRIM23, CLDN11, ABCA10, ETV5, TRIM2, TP53INP1, ST6GAL1, THBS2, ERAP1, OGT, RARRES1, CTDSPL* and *TBCC)* with highest significance for glycated haemoglobin (HbA1C) that is considered as the most chronic marker of hyperglycaemia. Gene *ST6GAL1* has already been identified as T2D-susceptibility locus [27] in a genome-wide association study in individuals of South Asian ancestry and SNPs at *ST6GAL1* were found to be associated with the pancreatic β-cell function. High expression of another gene *THBS2*, a potent anti-angiogenic protein, has been reported in high glucose condition-led oxidative stress and ascribed to diabetic bone marrow-derived angiogenic cell dysfunction [28]. We anticipate this panel of genes may be used as biomarkers for diagnosis of hyperglycaemia, particularly for Asian Indians.

## Discussion

The results of this study can be summarized as follows: (1) The femoral subcutaneous adipose tissue in diabetics showed pathologic changes in terms of adipose tissue hypertrophy and deregulation of inflammation-related pathways. (2) Several modules of co-expressed genes showed significant correlation with various diabetes-related intermediate phenotypic traits. Additionally, several genes in these modules were also differentially expressed in diabetics. In other words, these results support the occurrence of pathologic changes in peripheral subcutaneous adipose tissue, in diabetics and its association with some of the known aspects of the pathophysiology of insulin resistance.

Insulin resistance precedes β-cell dysfunction in the natural history of the disease and in Asian Indians its pathophysiology is expected to be unique for the given *lean fat* phenotype. Currently, the accumulation of ectopic lipid metabolites, activation of the unfolded protein response (UPR) pathway and innate immune pathways have all been implicated in the pathogenesis of insulin resistance [29]. Clinical and experimental evidence suggests that disordered remodelling of adipose tissue leads to adiposopathy and the consequently altered adipo-cytokinemia is the central mechanism of this ectopic lipid deposition. The cardinal features of the pathologic adipose tissue are adipocyte hypertrophy resulting from poor adipogenesis, poor angiogenesis, immune cell infiltration, changed macrophage polarity to pro-inflammatory M1 type and fibrosis.

In the present study, we attempted to illustrate the pathology of peripheral subcutaneous adipose tissue and its role in the pathogenesis of insulin resistance in terms of co-expressed genes showing the statistical correlation with intermediate traits of T2D and its associated disorders. As co-expression modules contain both statistically significant (*P < 0.05*) as well as insignificant DE genes (*P > 0.05*), we argue that even insignificant DE genes co-expressing with a good number of significant DE genes might have biologically important roles and therefore should be incorporated in the downstream functional analysis. We therefore, opted for the SPIA method, which is expected to retain the sensitivity and specificity of analysis due to the inclusion of pathway topologies. Results obtained from SPIA were also found to be in good agreement with the gender-wise pathway enrichment analysis. The results of this study substantiate our hypothesis that the protective fat portrays the classical picture of sick fat or adiposopathy otherwise, considered to be the hallmark of obesity and metabolic syndrome in T2D patients. Ours is probably the first report on the pathologic changes in femoral subcutaneous adipose tissue in Asian Indian diabetic patients. Even for Caucasians, there are only a few reports on whole genome expression profiling in gluteal-femoral adipose tissue. It is further emphasized here that among the various components of pathologic adipose tissue, it is the adipocyte hypertrophy (consequent to adipogenesis failure) that showed a positive correlation with clinical phenotypes like BMI, clinical diabetes, insulin resistance, leptin and negative correlation with β-cell function. Moreover, on *WGCNA* analysis five out of the seven modules (*turquoise, brown, green, red and blue*) showed a correlation with adipocyte size. Some of them also showed an association with diabetes phenotype itself and markers of inflammation such as hsCRP, and adipocytokine such as adiponectin. It is interesting to note that these modules enrich pathways related to adipogenesis, hypoxia, oxidative stress, angiogenesis failure, inflammation, cross-talk between adipocyte/adipokine and immune cells (like Toll-like receptor pathway). These findings are consistent with the theory of adiposopathy which states that under the conditions of calorie excess, adipogenesis failure leads to adipocyte hypertrophy, oxidative stress, adipose tissue hypoxia (due to large adipocyte size or angiogenesis failure). They all in turn lead to adipose tissue inflammation including a vicious cycle of Toll-like receptor activation on macrophages by heightened free fatty acid flux from hypertrophied adipocyte and cytokines released from these macrophages incite adipocyte inflammation. It is worth mentioning here that though transcription profile of adipose tissue revealed differential expression of inflammation-related pathways and all the modules of co-expressed genes enriched for inflammation-related pathways and their association with the markers of systemic inflammation like hs-CRP, leptin, TNF-α points towards the role of femoral subcutaneous adipose tissue in systemic inflammation and insulin resistance. However, we did not observe any significant change in the number of immune cells in adipose tissue (results not shown). Though there was a trend towards alteration in the cell type, relatively smaller sample size of this study restrains us from answering this question. Therefore, further investigations with a larger sample size are needed to resolve this issue.

In conclusion, the findings of the present study point towards the pathologic potential of femoral subcutaneous adipose tissue in Asian Indian diabetics. Additionally, the correlation of some of the modules of co-expressed genes with the disease state and its intermediate phenotypic traits highlights an association between this pathology and insulin resistance in them. This association could be causative or vice versa, therefore, requires further investigation. We further envisage that our *WGCNA*-based consensus network approach in conjugation with SPIA has successfully bridged genome-scale molecular events with clinical phenotypes.
